# A Test for the Purity of Idoform

**Published:** 1884-11

**Authors:** 


### A Test for the Purity of Iodoform.

Dr. Bouma, of Leyden, is of the opinion that the symptoms of iodoform poisoning are due to the presence of impurities in the drug used, and in the *Centralbl. f. Chir.* (Glasgow *Med. Jour.*) brings forward striking facts from the practice of several surgeons in proof of this. He holds that no iodoform should be used which has not been tested for impurities, and states that he has not yet seen unfavorable symptoms arise in a case where this rule has been adhered to. The most reliable test is a modification by H. Azema, apothecary to the Leyden Hospital, of one given by most authorities, and consists in shaking up the iodoform with distilled water, filtering, adding to the filtrate an alcoholic solution of nitrate of silver, and allowing this to stand for twenty-four hours. At the end of this time a slight grey deposit will usually be found at the bottom of the test tube; but anything approaching to a black precipitate (reduced silver) indicates that the iodoform is not pure enough to be used.

Dr. Bouma believes, however, that iodoform which has been kept for some time exposed to light and air may cause evil effects, even though this test fails to detect impurities.

An exchange says: "A widow shot herself in the oil regions the other day."

